# Outcome of Conservative Therapy of Adolescent Idiopathic Scoliosis (AIS) with Chêneau-Brace

**DOI:** 10.3390/jcm12072507

**Published:** 2023-03-26

**Authors:** Wojciech Pepke, William Morani, Marcus Schiltenwolf, Tom Bruckner, Tobias Renkawitz, Stefan Hemmer, Michael Akbar

**Affiliations:** 1 Department of Orthopaedics, Heidelberg University Hospital, 69118 Heidelberg, Germany; 2 Institute of Medical Biometry and Informatics, University of Heidelberg, 69120 Heidelberg, Germany; 3 Meoclinic, Friedrichstraße 71, 10117 Berlin, Germany

**Keywords:** adolescent idiopathic scoliosis, AIS, brace therapy, Cobb angle, Chêneau

## Abstract

Chêneau-brace (C-Brace) is a potential tool for the treatment of adolescent idiopathic scoliosis (AIS) with a Cobb angle between 20° and 45° for the primary curve. The aim of the present study was (1) to estimate study cohorts with C-brace therapy success and therapy failure and (2) to analyze possible factors that influence the therapy outcome. Seventy-eight patients with AIS were assessed before the initiation of C-brace treatment. Each patient underwent radiography examinations before the brace, in-brace, and at the therapy end. Cobb angle was considered as increased when the value at the end of therapy was increased more than 5° (Δ > 5°), unchanged—when the value was unchanged within ± 5° and decreased- when the value was decreased more than 5° (Δ < −5°). The study cohort was stratified due to curve topography in the thoracic, thoracolumbar, and lumbar scoliosis groups. Global analysis revealed no statistically significant modification of the Cobb angle (Cobb angle pre-brace vs. Cobb angle post-brace: 30.8° ± 8.2 vs. 29.3° ± 15.2, *p* = 0.26). However, at the end of C-brace therapy, the primary Cobb angle was decreased by more than 5° in 27 patients (35%), unchanged (Δ within the range of ±5°) in 36 patients (46%), and increased more than 5° in 15 patients (19%). Sub-group analysis due to curve topography and skeletal maturity has shown higher rates of brace therapy failure in thoracic curves and in younger patients (Risser grade 0). Patients with higher Cobb angle correction with C-brace had lower rates of therapy failure. The C-brace can be useful for the prevention of scoliotic curve progression in patients with AIS. However, many factors influence the therapy effect.

## 1. Introduction

Adolescent idiopathic scoliosis (AIS) is a complex deformity of the spine in all three planes arising in otherwise normal children during puberty. There is a number of different pathogenetic theories that were discussed as a possible cause for AIS onset. These factors can be classified into intrinsic and extrinsic factors. The role of genetics in the onset of AIS has been described by several authors [1,2,3,4,5], however, the specific mode of heredity transmission is still not resolved. Secondly, asymmetrical growth of the vertebrae was considered a possible etiologic factor in the genesis of AIS. Due to the established law of Hueter and Volkmann, an asymmetric load of the epiphysial vertebral plates can result in differing growth and vertebral wedging [6]. Also, disturbance of posterior column growth resulting in hypokyphosis or even lordosis of the thoracic spine by relative anterior vertebral overgrowth was discussed as a possible genesis of AIS [7,8,9]. The fully erect posture with an axial vertebral load while standing position, which is unique in humans, might be a precondition for the onset of AIS [10]. On the other side, there is no assured cause for the onset of AIS so far and its development seems to be multifactorial [11].

Since the report of the Scoliosis Research Society (SRS) in 1982, it is known that 2 to 3% of adolescent people younger than 16 years of age will have scoliotic curvature of 10°, but only up to 0.5% will evolve a curvature of 20° [12]. The clinical relevance of scoliosis on disability and quality of life is already proven. Long-term follow-ups reveal that scoliosis patients may have a higher prevalence of back pain and disorder of pulmonary function in case of large curve progression [13]. For that, the aim of therapy of AIS is to prevent life quality impairment during adulthood by stopping the curve progression in adolescence.

The treatment modalities of AIS range from conservative procedures such as physiotherapy and sports, brace therapy, to surgical procedures [14]. Brace treatment is an established cornerstone of non-operative management that aims to stop deformity progression before reaching the threshold of indication for surgery. The Chêneau-brace (C-brace) belongs to rigid thoraco–lumbo–sacral orthoses (TLSO) and is most widely prevalent in Europe [15]. Using multiple pressure areas and expansion chambers, C-brace intends to handle the scoliotic curves in coronal, sagittal and transversal dimensions [15,16]. The effectiveness of bracing in the treatment of AIS is not controversial anymore. Former studies carried out that brace therapy can stop or diminish the progression of scoliosis and presumably help to avoid surgery [17,18]. Certainly, current quality standards of brace manufacturing have to be applied to achieve good clinical results [19,20].

However, published data about the effectiveness of bracing in the treatment of AIS is still needed. A previous Cochrane systematic review (2010) revealed low-quality evidence in reference to brace therapy [21]. The same research group revealed in their review of seven studies due to brace therapy for AIS that bracing avoids curve progression. On the other hand, low and moderate methodological quality of these studies remains a solid estimation of the brace effectiveness uncertain [22]. Lorenzo et al. could show in their meta-analysis that the majority of brace studies have a significant risk of bias, but bracing therapy is effective for AIS treatment [23]. The statement about the effectiveness of brace treatment is probably too general, which raises questions regarding the relevance of such a statement for each individual with AIS [24]. The purpose of this study was (1) to estimate study cohorts with C-brace therapy success and therapy failure and (2) to analyze possible factors that influence the therapy outcome.

## 2. Materials and Methods

### 2.1. Study Cohort

This is a retrospective and single-center study of conservatively treated AIS patients with a Cobb angle between 20° and 45°, in whom the therapy was started between 2010 and 2018. Individuals were included with Risser grades from 0 to 2. All patients had no brace therapy before and were adolescents at the beginning of C-brace treatment. Further inclusion criterium was the existence of full spine radiographs in anterior-posterior and lateral view before brace, with a brace and at follow-up. Exclusion criteria were diagnosis of early onset scoliosis, congenital, neuromuscular, and syndromic scoliosis (due to performed therapy with other brace types). Patients with insufficient image quality on radiographs were excluded. All patients that fulfilled the inclusion criteria, were included in this study.

The whole study cohort was treated in one institution using only one type of brace fabricated by one experienced brace maker. The usual duration of brace manufacturing lasted about three months (81 ± 27 days). After the fabrication of the C-brace and validation with in-brace radiographs, the therapy was conducted for one year. All patients were instructed to wear the C-brace for 23 h per day. After six months, clinical controls were performed due to brace fitting accuracy in growing patients. After one year, new radiographs of the whole spine without brace were performed to prove the efficacy of the treatment. In the case of still present Risser grade from 0 to 2, a new C-brace was manufactured for further therapy until the Risser grade 3 was reached.

Adolescents with AIS and C-brace therapy got a recommendation of physiotherapy as an additive therapy to brace. The physiotherapeutic treatment for AIS patients involves back therapy training and physiotherapeutic scoliosis-specific exercises (PSSE). The recommended frequency for long-term physiotherapeutic sessions was two to three times per week if patients were willing to cooperate. The form of exercises depended mainly on the character of therapeutic methods chosen by the physiotherapist and patient. For the duration of all sports activities and personal hygiene it was allowed to take C-brace off.

This study was approved by the ethics committee of Heidelberg University (permission No. S-196/2019).

### 2.2. Data Collection and Analysis

**Figure 1:**
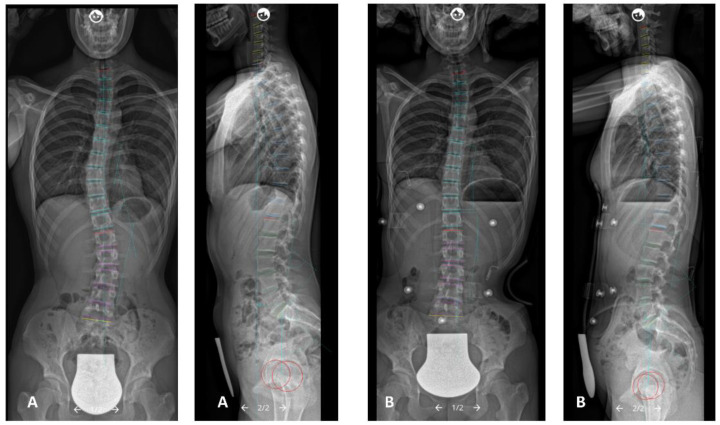
(**A**) Pre- and (**B**) in-brace anterio-posterior and lateral radiographs illustrating the analyzed spinal parameters (measurements with Surgimap software (Surgimap^®^, New York, NY, USA)).

Full spine radiographs were acquired using a low-dose biplanar EOS-imaging device (EOS-imaging^®^, Paris, France) or conventional radiography. Radiographic controls were performed before the brace, in-brace, and at follow-up after finished brace therapy. First, in-brace radiographic controls were performed after completing the fabrication process of the C-brace. In all radiographic controls, all patients had their hands on their cheeks to avoid the upper extremities overlapping the spine [25], were barefoot, and were instructed to look straight ahead. Patients, whose radiographs that did not pass the mentioned requirements were not included. Data were stored as a digital imaging and communications in medicine file (DICOM). Analysis of the radiographic controls was conducted with Surgimap software (Surgimap^®^, New York, NY, USA) [26] (Figure 1). Radiographic measurements were performed by a single reader (W.M.), a research fellow and consultant for spine surgery. Radiographic parameters included the following:

Coronal parameters: primary Cobb angle for main curve, secondary Cobb angle for compensatory curve, coronal alignment (Calignment), C7-plumbline (C7PL).

Sagittal spinopelvic parameters: T1–T12 thoracic kyphosis TK (T1–T12), T4–T12 thoracic kyphosis TK (T4–T12), L1-S1 lumbar lordosis (LL), pelvic incidence (PI), pelvic tilt (PT), sacral slope (SS), C7-S1 sagittal vertical axis (SVA), T1-spinopelvic inclination (T1 SPi), T9-spinopelvic inclination (T9 SPi).

Axial plane parameters: apical vertebral rotation (AVR) of the primary curve (Raimondi 1). Raimondi rotation angle is an established method for the measurement of vertebral rotation in standard radiographs of the spine [27].

### 2.3. Patient Stratification for Sub-Group Analysis

Patients of the study cohort were divided due to their scoliosis topography into three groups: (1) thoracic curves with coronal apex between T3 and T9, (2) thoracolumbar curves—apex between T10 and L1, and (3) lumbar curves—apex between L2 and L4.

### 2.4. Statistical Analysis

Statistical analysis was performed with SPSS^®^ Software (IBM^®^, Armonk, NY, USA, Version 25). Data are presented as mean ± standard deviation (SD). Paired *t*-test was used for intergroup comparisons. The threshold for statistical significance was defined at *p* < 0.05. Absolute values of the coronal plane and axial vertebral rotation were used for the analysis of the severity of coronal deviation and axial rotation, but without considering the direction. Due to the retrospective study design and inclusion of all subjects that fulfilled inclusion criteria, the performance of power analysis was not mandatory.

## 3. Results

### 3.1. Global Analysis

**Figure 2:**
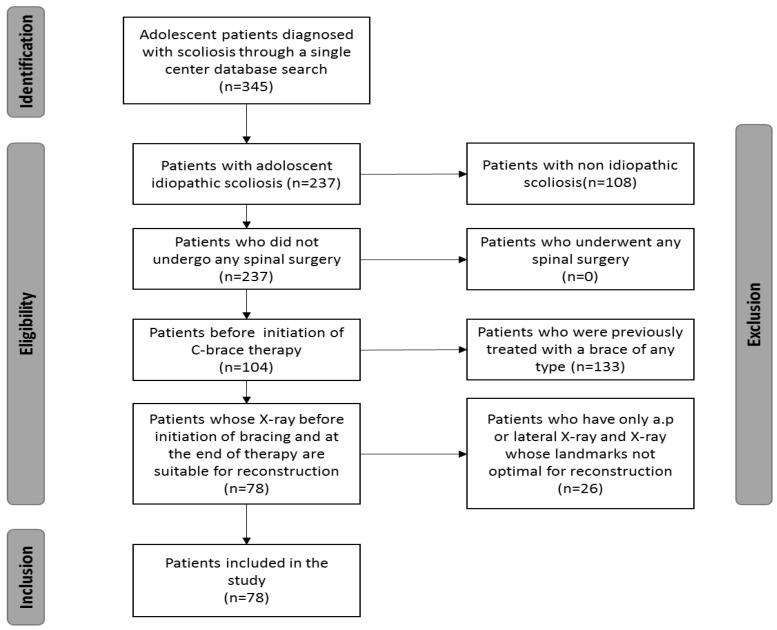
Diagram with inclusion and exclusion process.

**Figure 3:**
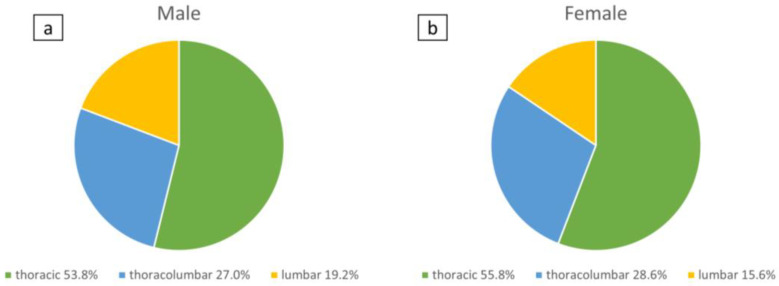
Distribution of study cohort males (**a**) and females (**b**) in subgroups regarding curve topography (thoracic, thoracolumbar, and lumbar scoliosis).

A total of seventy-eight patients (74% females, n = 58; 26% males, n = 20) with a mean age of 12.8 ± 2.1 years were included in the study. A total of 35% (n = 27) had a Risser grade 0, 27% (n = 21)—Risser grade 1, and 38% (n = 30)—Risser grade 2. A total of 56% of the study population had thoracic scoliosis (n = 44), 27% of patients had thoracolumbar scoliosis (n = 21), and 17% of patients had lumbar scoliosis (n = 13). Figure 2 shows the inclusion and exclusion process. The distribution of males and females was in all three subgroups regarding the topography of scoliotic curves similar as shown in Figure 3.

**Figure 4:**
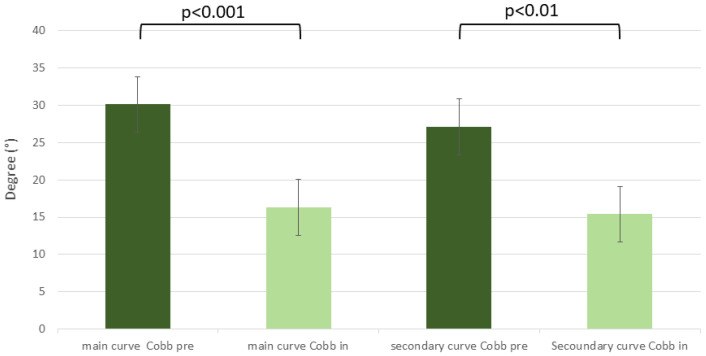
Cobb angle for primary and secondary curves before and with C-brace.

While C-brace therapy, in-brace measurements of the whole study population revealed significant adjustment of primary and secondary Cobb angles as shown in Figure 4.

Comparison of coronal and sagittal parameters of the whole study population (regardless of the curve type) before C-brace therapy and at the final follow-up revealed no significant changes in Cobb angle (Table 1). Merely, TK1 was slightly but significantly flattened (TK1 pre brace vs. TK1 post therapy: 30.8 ± 8.2 vs. 29.3 ± 15.2; *p* = 0.02). All other measured sagittal parameters and Raimondi angle for the axial plane were unchanged.

### 3.2. Sub-Analysis by Increased, Unchanged, and Decreased Cobb Angle

Although global analysis of primary and secondary Cobb angle revealed no significant changes before and at the end of the C-brace therapy, further sub-analysis of the study cohort determined some important findings due to measured parameters. At the end of C-brace therapy, the primary Cobb angle was decreased by more than 5° in 27 patients (35%), unchanged (change within the range of ±5°) in 36 patients (46%), and increased more than 5° in 15 patients (19%). The variation of other parameters in this sub-analysis is shown in Table 2.

**Figure 5:**
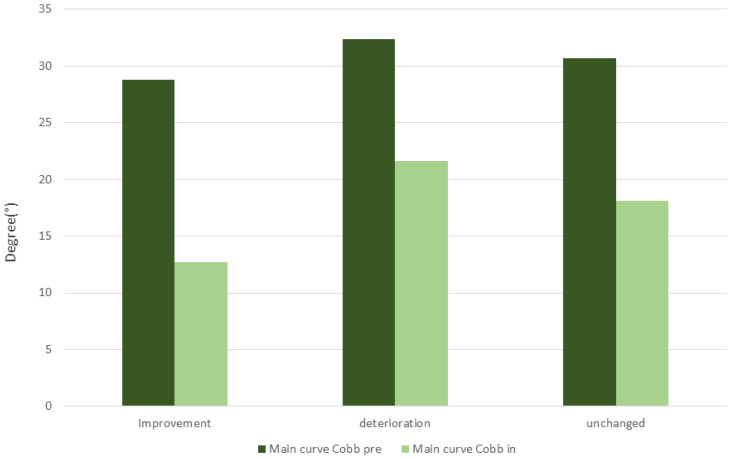
In-brace Cobb angle correction for group improvement, unchanged, and deterioration of primary Cobb angle at the end of C-Brace therapy.

Sub-analysis of the in-brace primary Cobb angle in comparison to the pre-brace primary Cobb angle revealed differences between all three groups. The group with improved primary Cobb angle at the end of C-brace therapy, had the most in-brace Cobb angle correction for the time of therapy. The group with the finally unchanged Cobb angle had moderate in-brace Cobb angle correction, and the group with the final deterioration of the primary Cobb angle, the fewest in-brace Cobb angle correction, as shown in Figure 5.

**Figure 6:**
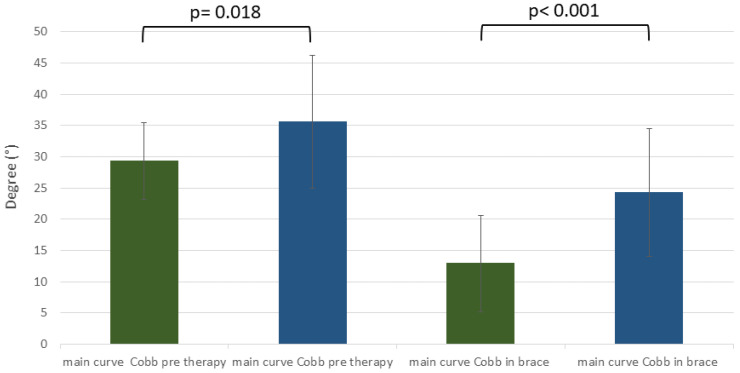
Before brace and in-brace Cobb angle of improvement group (green) and deterioration group (blue).

Furthermore, the comparison of the improvement and deterioration groups revealed significant difference due to the primary Cobb angle before C-brace therapy (primary Cobb angle before brace in the improvement group vs. primary Cobb angle before brace in deterioration group: 29.3° ± 6.1 vs. 35.6° ± 10.6, *p* = 0.018) (Figure 6). Also, the in-brace primary Cobb angle in the deterioration group was significantly higher than in the improvement group (primary Cobb angle in-brace in improvement group vs. primary Cobb angle in-brace in deterioration group: 12.9° ± 7.7 vs. 24.3° ± 10.2, *p* = <0.001) (Figure 6).

The distribution of males and females was in all three subgroups similar and corresponded to the whole study cohort. In the improvement group there were 29% males and 71% females, in the unchanged group—17% males and 83% females, and in the deterioration group—33%males and 67% females.

**Figure 7:**
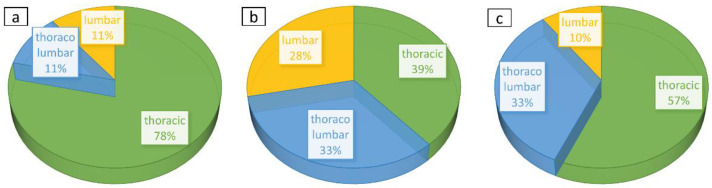
Diagrams illustrating percentage distribution due to topography of scoliosis in the study cohort. (**a**)—deterioration group, (**b**)—improvement group, (**c**)—unchanged group.

Interestingly, the study of the topography of the scoliotic curves in defined subgroups showed some differences between them. In the group with primary Cobb angle deterioration after C-brace therapy, there was an overwhelming part of thoracic scoliosis patients (78%). In opposite to that, the group with post-therapeutic primary Cobb angle improvement, was represented by a higher part of individuals with thoracolumbar (33%) and lumbar (28%) scoliosis as shown in Figure 7.

**Figure 8:**
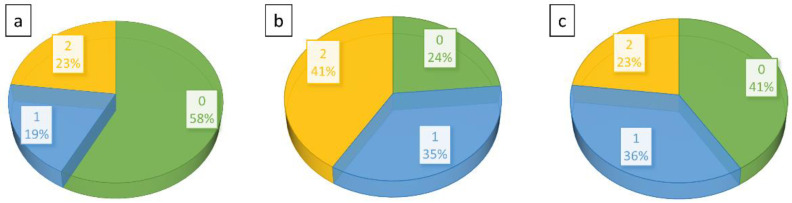
Diagrams illustrating percentage distribution of skeletal maturity measured with Risser grade (0 to 5) in the study cohort. (**a**)—deterioration group, (**b**)—unchanged, and (**c**)—improvement group.

The study of skeletal maturity of the subgroups revealed a higher part of individuals with Risser grade 0 (58%) in the primary Cobb angle deterioration group as in the primary Cobb angle improvement group (41%) as shown in Figure 8.

## 4. Discussion

The instant correction of AIS in the coronal, sagittal, and transversal plane induced by C-brace treatment has been delineated [28,29]. The purpose of this research was to analyze the outcome variability after C-brace therapy in AIS patients.

The main expected goal of C-brace therapy is to hold the shape of the scoliotic spine and to avoid curve progression. In our study cohort, the success rate of C-brace therapy (all subjects with improved or unchanged primary Cobb angle) was by 81%. Fang et al. showed in their patients series of similar successful rates of C-brace therapy [15]. In related research, success rates associated with other types of brace were lower than those associated with C-brace [30,31,32] indicating good results in preventing Cobb angle progression in our study cohort. The results of this research differed from the results of De Giorgi et al. [33], who reported a higher rate of Cobb angle correction for all patients at the final follow-up. However, the study of Giorgi et al. may have a bias due to the small study cohort. In summary, the results of this study demonstrate that it is possible to obtain satisfactory prevention of coronal curve progression, when treatment was started at the appropriate time and C-brace manufacturing was adequate.

Former studies of the efficacy of brace treatment were focused on coronal curves and reports about sagittal profile changes were sparse [15,28,34]. Within the few last years there was a focus also on the sagittal profile that could be altered through bracing [35,36,37]. This phenomenon of flattening of TK and LL and also slight changes of pelvic and cervical parameters while brace therapy (in-brace measurements) we reported in former studies [29,38]. In our research, the brace-induced changes of pelvic, lumbar, and thoracic parameters were variable and in global analysis with exception of TK unchanged. Only TK1 (TK T1/T12) was at the end of brace therapy significantly flattened (*p* = 0.02). Nevertheless, progressive changes in the spine parameters may appear throughout the treatment period. Interestingly, although the PI of the whole study population was unaltered, in a few subjects the PI was changed over a mark of 5° at the end of brace therapy. Previous studies delineated that PI is a widely fixed value. However, Mac-Thiong et al. and others [39,40,41] reported about a marginal tendency for PI to change after the acquisition of bipedalism during growth until skeletal maturity. The question is if C-brace pads and their long-term pressure on the pelvis may cause changes in PI in young patients as described by Fang et al. [15].

Sub-analysis of the study cohort due to primary Cobb angle improvement or deterioration while C-brace therapy revealed further important findings. In this study, AIS patients with primary Cobb angle improvement at the end of therapy, had a higher Cobb correction (Δ) while C-brace therapy, as patients with unchanged Cobb angle and patients with Cobb angle deterioration as shown in Figure 5. First, in-brace correction is the result of the initial brace application which depends on the flexibility of the primary curvature. Former studies revealed that insufficient in-brace correction is a predictive factor for therapeutic failure while using a brace [42,43,44]. Despite reported variability between studies due to the magnitude of needed in-brace correction, this study revealed that AIS patients with improved first in-brace correction are less prone to treatment failure. Goodbody et al. reported that Cobb angle correction with a brace less than 45% is associated with treatment failure (*p* < 0.001) [45]. We consider our outcomes for brace treatment to be in line with previous studies’ findings [42,43].

Also, the initial primary Cobb angle seems to play a role as a risk factor for curve progression. The comparison of sub-groups with Cobb angle improvement and Cobb angle deterioration revealed a significant difference due to Cobb angle magnitude (*p* = 0.018) before the start of C-brace therapy. Interestingly, the mean primary Cobb angle in the deterioration group was >30°, and in the improvement group <30°. These findings are in line with the results of other research. In summary, six papers covering a total of 912 individuals with AIS identify an initial Cobb angle of >30° to 40° as an important risk factor for curve progression despite brace therapy [46,47,48,49,50]. Another important factor relates to skeletal maturity at the time of indication for C-brace. There was a relevant difference in relation to Risser grade distribution in the improvement and deterioration group. A higher rate of Risser grade 0° was noticed in the deterioration group (58%) and a relatively lower rate in the improvement group (41%). This indicates that skeletal maturity might have an influence as a prognostic factor on scoliotic curve progression or improvement while undergoing C-brace therapy, as already postulated by other authors. Charles et al. demonstrated that untreated AIS patients with Risser grade 0 with primary curve magnitude > 30° at the onset of the accelerating growth phase will progress to more than a 45° curve magnitude to 100% of the time [50]. Karol et al. concluded that in general patients with Risser grade 0 and a curve of ≥30° are predisposed to progress to surgery even when the brace is being used [47].

In this study cohort, sub-analysis revealed further important aspects for efficacy of C-brace therapy. Due to the topography of scoliotic curves, we identified that the rate of failure is higher in individuals with the thoracic main curve compared to in patients with the thoracolumbar or lumbar primary curve. The reason for the better success rate in thoracolumbar and lumbar scoliosis patients may be the morphology of scoliosis and biomechanics of the C-brace [29,51]. In thoracic curves, the positioning of the upper pad may be difficult owing to the arms of the AIS patients. Furthermore, a bony thorax may hinder good correction [51].

Overall, the failure rate in this study was acceptable in comparison to the results of other authors. A strength of this study was that one brace type was used from the same experienced brace maker, which should decrease the bias related to the mixing of different designs of a brace from different institutions [33].

Limitations of the present study included the low number of patients, retrospective study design, and the absence of a control group. However, ethical considerations about unessential radiographs in healthy volunteers or non-application of C-brace in AIS patients with indication for brace were prohibitive. Secondly, the application of Lenke classification [52] was impossible due to small sub-groups. Moreover, the brace maker’s experience is essential to achieving good results. It might be a strong point that the whole study cohort was treated in one institution using only one type of brace being fabricated by one experienced brace maker [33]. However, we acknowledge this as a limitation. The handcraft of brace makers is crucial to enhancing the efficacy of brace therapy and hence would bias the discussed results [15,53]. Furthermore, the lack of further controls after finished C-brace therapy should be considered a limiting factor. This point should be addressed, as soon as a sufficient amount of post-therapy long-term radiographic controls is available for analysis. Finally, software measurement error also has to be considered. However, the software used in this study is validated and a high-reliability level of the computerized measurements was already proven [26].

## 5. Conclusions

The main goal of the C-brace therapy of AIS patients is to provide coronal curve correction without impairment of the sagittal profile. The results of this study implicate that C-brace therapy can be effective in the prevention of curve progression during a growth spurt. However, our results reveal the high efficacy variability due to factors such as primary Cobb angle magnitude, skeletal maturity, and in-brace Cobb angle correction at the begin of C-brace therapy. The study results might shed some light on AIS patients’ treatment in context of bracing and might be beneficial for treating physicians.

## Figures and Tables

**Table 1:** Pre-brace to post-therapy changes of coronal and sagittal parameters regardless of the curve type.

Parameter	Before Treatment	At Final Follow-Up	Difference	95%CI	*p* Value
Cobb angle (°)	30.8 ± 8.2	29.3 ± 15.2	1.5 ± 11.2	−1.0 to 3.9	0.26
TK1 (°)	33.7 ± 13.7	31.2 ± 12.4	−2.5 ± 9.4	0.3 to 4.5	0.02
TK2 (°)	26.7 ± 13.8	25.8 ± 12.3	−0.8 ± 8.9	−1.1 to 2.9	0.38
LL (°)	57.9 ± 12.1	56.0 ± 12.7	1.9 ± 9.4	−4.0 to 0.1	0.07
PT (°)	7.8 ± 8.3	8.9 ± 7.9	1.0 ± 7.5	−2.7 to 0.5	0.20
PI (°)	50.6 ± 14.5	50.8 ± 14.3	0.2 ± 2.5	−0.7 to 0.3	0.49
SS (°)	42.3 ± 10.0	41.1 ± 9.9	−1.1 ± 6.9	−0.3 to 2.7	0.13
T1SPi (°)	−4.3 ± 3.9	−4.5 ± 2.3	−0.1 ± 4.0	−0.6 to 1.1	0.64
T9SPi (°)	−6.2 ± 4.7	−6.6 ± 3.2	−0.4 ± 4.5	−0.6 to 1.4	0.42
Raimondi 1 (°)	17.6 ± 9.4	18.0 ± 9.6	0.3 ± 9.7	−2.5 to 1.8	0.76
SVA (mm)	−10.1 ± 32.7	−10.0 ± 22.9	0.2 ± 33.3	−7.5 to 0.9	0.98

Value is presented as the mean ± SD. TK1—(T1/T12) thoracic kyphosis, TK2—(T4/T12) thoracic kyphosis, LL—(L1/S1) lumbar lordosis, PT—pelvic tilt, PI—pelvic incidence, SS—sacral slope, T1SPi—T1 spinopelvic inclination, T9SPi—T9 spinopelvic inclination, SVA—sagittal vertical axis.

**Table 2:** Cobb angle for primary and secondary curves before and with C-brace.

No. of Patients (%)												
	Coronal Parameters		Sagittal Parameters					Axial Rotation	Pelvic Parameters			
Variation	Cobb Angle	CorC7PL	TK1	TK2	LL	C1/C2	T1 Slope	Raimondi 1	PT	PI	SS	SVA
Increased	15 (19)	13 (17)	18 (23)	23 (29)	29 (37)	11 (14)	18 (23)	22 (28)	17 (22)	2 (2)	14 (18)	32 (41)
Unchanged	36 (46)	33 (42)	34 (44)	34 (44)	35 (45)	35 (45)	28 (36)	38 (49)	47 (60)	72 (92)	50 (64)	14 (18)
Decreased	27 (35)	32 (41)	26 (33)	21 (27)	14 (18)	32 (41)	32 (41)	18 (23)	14 (18)	4 (5)	14 (18)	32 (41)

A parameter was considered increased when the value at the end of therapy was increased >5° or >25 mm (SVA) over that at initiation, unchanged when the value was <5° or <25(SVA) mm over that at initiation, and decreased when the value decreased >5° or >25 mm (SVA) from that at initiation. CorC7PL—coronal C7 plumbline, TK1—(T1/T12) thoracic kyphosis, TK2—(T4/T12) thoracic kyphosis, LL—(L1/S1) lumbar lordosis, C1/C2—C1/C2 angle, Raimondi 1—axial rotation of the apical vertebra, PT—pelvic tilt, PI—pelvic incidence, SS—sacral slope, SVA—sagittal vertical axis.

## Data Availability

The datasets used and analyzed during the current study are available from the corresponding author on reasonable request.
